# Past and future of trypanosomatids high-throughput phenotypic screening

**DOI:** 10.1590/0074-02760210402

**Published:** 2022-03-11

**Authors:** Rafael Ferreira Dantas, Eduardo Caio Torres-Santos, Floriano Paes Silva

**Affiliations:** 1 Fundação Oswaldo Cruz-Fiocruz, Instituto Oswaldo Cruz, Laboratório de Bioquímica Experimental de Computacional de Fármacos, Rio de Janeiro, RJ, Brasil; 2 Fundação Oswaldo Cruz-Fiocruz, Instituto Oswaldo Cruz, Laboratório de Bioquímica de Tripanosomatídeos, Rio de Janeiro, RJ, Brasil

**Keywords:** trypanosomatids, phenotypic, high-content screening, bioimaging, drug discovery

## Abstract

Diseases caused by trypanosomatid parasites affect millions of people mainly living in developing countries. Novel drugs are highly needed since there are no vaccines and available treatment has several limitations, such as resistance, low efficacy, and high toxicity. The drug discovery process is often analogous to finding a needle in the haystack. In the last decades a so-called rational drug design paradigm, heavily dependent on computational approaches, has promised to deliver new drugs in a more cost-effective way. Paradoxically however, the mainstay of these computational methods is data-driven, meaning they need activity data for new compounds to be generated and available in databases. Therefore, high-throughput screening (HTS) of compounds still is a much-needed exercise in drug discovery to fuel other rational approaches. In trypanosomatids, due to the scarcity of validated molecular targets and biological complexity of these parasites, phenotypic screening has become an essential tool for the discovery of new bioactive compounds. In this article we discuss the perspectives of phenotypic HTS for trypanosomatid drug discovery with emphasis on the role of image-based, high-content methods. We also propose an ideal cascade of assays for the identification of new drug candidates for clinical development using leishmaniasis as an example.

*Trypanosomatids and neglected tropical diseases* - Trypanosomatids (Euglenozoa: Kinetoplastea) are a group of protozoan obligatory parasites.^1^ ^,^ ^2^ Most members of this group are monoxenous (single host parasites) and infect invertebrates.^3^ However, some dixenous (parasites with two intermediate hosts) species act as etiological agents of neglected tropical diseases, such as Chagas disease (*Trypanosoma cruzi*), African trypanosomiasis (*T. brucei*) and human leishmaniasis (more than 20 species).^2^ ^,^ ^3^ ^,^ ^4^ Here, we will mainly focus on the *T. cruzi* and species from the *Leishmania* genus.

Recent estimates suggest that 6 to 7 million people worldwide may be infected with *T. cruzi*, maily in Latin America, and 75 million are at risk of infection.^5^ The classic route of transmission to humans is through hematophagous triatomine bugs infected with the parasite. This occurs during or right after the blood meal when the insect defecates on host skin. Its feces contain the metacyclic trypomastigote evolutionary form of *T. cruzi* which is able to penetrate the skin through the wound bite, other skin lesions or mucous membranes. Once inside the host, the parasites infect numerous types of cells, especially those from the reticuloendothelial system, muscular and nervous cells. After the infection, the parasites differentiate into amastigote forms which proliferate by binary fission. After several replication cycles, they evolve into trypomastigotes that disrupt the cell and reach the bloodstream, allowing them to invade other cells in the organism or be taken up by another triatomine bug, continuing the parasite life cycle.^6^

*Trypanosoma cruzi* infection is responsible for the clinical outcomes of Chagas disease ranging from no apparent symptoms to severe and potentially deadly cardiovascular and/or gastrointestinal manifestations.^7^ This variability has been associated with factors related to both host and parasite.^8^ One possible explanation may be derived from parasites’ genetic background. These parasites show a high genetic variability being assembled into seven distinct genetic groups, or discrete typing units (DTU): TcI-VI and TcBat.^9^ All of them can infect humans and their frequency varies depending on the geographic location.^8^ ^,^ ^9^ The link between *T. cruzi* genotype and the clinical manifestations (or drug susceptibility) of Chagas disease has been proposed but has not been proved yet.^6^ ^,^ ^7^ ^,^ ^9^ So far, there is no vaccine available and only two drugs, benznidazole (**1**) and nifurtimox (**2**), are clinically used. Nonetheless, they have several limitations, such as long treatment regimes, undesirable side effects (e.g., nausea, severe dermatitis and peripheral neuropathies) and clinical failure is not uncommon.^6^

The trypanosomatids from the *Leishmania* genus are the causal agents of one of the most devastating infectious diseases of our time: leishmaniasis.^10^ This disease affects millions of people worldwide and it is estimated that more than 1 billion are at risk of infection.^4^ Leishmaniasis is encountered in three main clinical forms: cutaneous (CL), visceral or kala-azar (VL) and mucocutaneous (MCL). The former is the most common while VL the most severe (fatal in more than 95 % of cases if left untreated) and MCL the most disabling.^11^ Each form is elicited by a group of *Leishmania* species which include: *L. major*, *L. tropica*, *L. braziliensis*, *L. amazonensis*, *L. guyanensis* for CL, *L. donovani* and *L. infantum* (also known as *L. chagasi*) for VL and *L. braziliensis*, *L. panamensis* and *L. amazonensis* for MCL.^12^ Regardless of the species, in most cases the parasites are transmitted to humans by the bite of infected female phlebotomine sand flies. During the blood meal, the insect’s saliva and the metacyclic promastigote form of the parasite are inoculated into the host. The latter induces a phagocytic response which allows the parasite to enter the macrophage (or other mononuclear phagocytic cells) and form the parasitophorous vacuole. Inside this compartiment, it differentiates into proliferating amastigote forms. Part of these infected cells can be taken up by the insect in another blood meal helping to maintain the parasite life cycle. In the human host, the continuous proliferation of amastigotes eventually leads to cell disruption and consequently release of the parasites, allowing them to infect other cells.^13^ ^,^ ^14^

*Leishmania* infection is responsible for the clinical features of leishmaniasis. For VL, the most fatal form of this disease, they include persistent irregular fever, splenomegaly, pancytopenia, hepatomegaly, and hyperpigmentation of the skin (hence the name kala-azar which can be translated as “black fever”). The CL form is not life-threatening but can lead to significant cosmetic morbidity due to the scars that arise after the healing of chronic skin lesions.^15^ ^,^ ^16^ In turn, MCL is mainly characterised by the presence of ulcers in the nasal septum, lips and palate. As for VL, this form may lead to death if not treated rapidly.^16^ Currently, there is no vaccine available, and the pharmacological treatments rely on a few drugs: pentavalent antimonials e.g., meglumine antimoniate (**3**), amphotericin B (**4**), pentamidine (**5**), paromomycin (**6**) and miltefosine (**7**) (Fig. 1). In most cases, the treatment is very broad and does not take into the account the peculiarities of each species. These drugs also have some major drawbacks, such as high cost, significant toxicity, must be administered via parenteral route except miltefosine (**7**) and may induce resistance.^10^

**Fig. 1:**
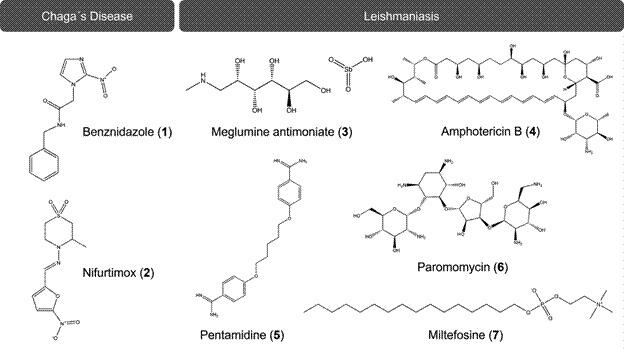
clinical drugs used in the treatment of Chagas disease and leishmaniasis.

*Phenotypic-based assays in trypanosomatid drug discovery* - The limitations of the current anti-trypanosomatid agents demand the search for new pharmacological alternatives. In this context, phenotypic-based assays play a pivotal role in trypanosomatid drug discovery.^17^ ^,^ ^18^ ^,^ ^19^ Most traditional methods use manual microscopy techniques (e.g., giemsa staining^20^ ^,^ ^21^ ^,^ ^22^ ^,^ ^23^) to evaluate the effect of a given compound on the number (amastigotes) or presence/motility (promastigotes) of the parasites.^12^ ^,^ ^24^ For instance, during a typical assay using intracellular amastigote forms, a microscope operator visually counts the number of host cells (100-500 per sample^25^) and intracellular parasites. From this analysis it is possible to calculate the percentage of infected cells (infection ratio), as well the average number of parasites per cell, which are used as metrics to measure compound antiparasitic activity.^20^ ^,^ ^21^ ^,^ ^22^ ^,^ ^25^ ^,^ ^26^ As expected, these methods are semi-quantitative, have low throughput and are prone to human errors. In an attempt to overcome these limitations, several phenotypic assays have been developed using more automated technologies such as, microplate readers, flow cytometers and high-content microscopes (discussed in the next section)^12^ ^,^ ^19^ ^,^ ^27^ (Fig. 2). The most common methods rely on fluorometric, luminescence or colorimetric readouts to measure parasites viability/growth in microplate readers, which are available in many laboratories. Trypanosomatids viability has been mainly assessed by measuring parasites ATP^28^ ^,^ ^29^ ^,^ ^30^ ^,^ ^31^ content and/or by measuring the metabolic reduction of redox probes, such as tetrazolium salts (MTT/XTT/MTS)^32^ ^,^ ^33^ ^,^ ^34^ and resazurin.^35^ ^,^ ^36^ ^,^ ^37^ The number of parasites has also been indirectly obtained by SYBR Green,^38^ ^,^ ^39^ a fluorescent nuclear probe. Some assays use transgenic parasites carrying fluorescent reporter genes e.g., GFP^40^ ^,^ ^41^ ^,^ ^42^ and mCherry^43^ or reporter enzymes e.g., beta-galactosidase,^41^ ^,^ ^44^ ^,^ ^45^ ^,^ ^46^ beta-lactamase,^47^ ^,^ ^48^ luciferase^49^ ^,^ ^50^ ^,^ ^51^ ^,^ ^52^ whose activities can be readily detected in the presence of their substrates.

**Fig. 2:**
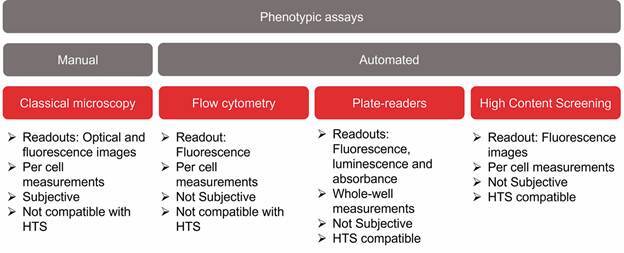
main phenotypic assays used in trypanosomatid drug discovery.

Although plate-reader-based assays represent a major advance in trypanosomatid drug discovery, they also show some important drawbacks. Firstly, they perform whole-well readouts which give no information regarding the number of host cells and parasites or their distribution.^53^ Some of them are also prone to assay interference. For instance, coloured compounds may affect the readout of colorimetric assays.^54^ Special attention should also be paid to hits coming from enzyme reporter-based assays since, theoretically, compounds may interfere with enzyme activity (or its substrate) and vice-versa, generating spurious results.^44^ ^,^ ^53^ ^,^ ^54^ Moreover, many assays were developed to test compounds against promastigotes^12^ ^,^ ^30^ ^,^ ^32^ ^,^ ^33^ ^,^ ^55^ and axenic amastigotes (i.e., amastigotes that are growth in culture media that simulate intracellular conditions)^29^ ^,^ ^53^ ^,^ ^56^ evolutionary forms. Though promastigotes are easy to handle and can be obtained in large amounts, which is desirable for HTS campaigns, they represent the vector-transmitted form of the parasite life cycle which is not directly involved in disease development.^38^ ^,^ ^57^ ^,^ ^58^ In turn, axenic amastigotes share more similarities with the intracellular forms, thus being more biologically relevant, and they have already been proved to be useful in library screening.^29^ ^,^ ^53^ Nonetheless, phenotypic assays based on both promastigotes and axenic amastigotes fail to some extent to identify active compounds (or reproduce their potency) on parasite intracellular forms and may generate false positives.^29^ ^,^ ^59^ ^,^ ^60^ In part, this is because these methods are unable to mimic the complex interaction between the parasite and the host cell.^29^ Additionally, in order to exert its antiparasitic activity, a compound must overcome some obstacles before reaching its targets which include transpassing several cellular membranes and coping with pH changes.^53^ ^,^ ^61^ ^,^ ^62^

Therefore, even lacking the throughput of plate-reader-based assays, the intracellular amastigote-based assays are still considered the gold standard for trypanosomatid drug discovery.^27^ ^,^ ^29^

*High content screening in trypanosomatid drug discovery* - In the late 90’s, a new generation of automated fluorescent microscopes emerged in the phenotypic drug discovery scenario.^63^ This technology, also known as high-content screening (HCS), automatically extracts multiparametric data, at a single-cell level, from fluorescent microscopy images acquired in a high-throughput mode.^63^ ^,^ ^64^ HCS systems offer individual, spatial and temporal information which can be applied in different stages of the drug discovery pipeline.^65^ ^,^ ^66^ Thus, HCS-based assays have been employed in a wide range of applications.^66^ In the context of trypanosomatid drug discovery, most reports use HCS technology to evaluate the effect of test compounds on intracellular amastigotes (Table). In these assays, host cells and parasites, distributed in microplates, are incubated with test compounds (one or multiple concentrations for dose-response curves), stained with one^67^ ^,^ ^68^ ^,^ ^69^ ^,^ ^70^ or more^58^ ^,^ ^71^ ^,^ ^72^ ^,^ ^73^ ^,^ ^74^ fluorescent probes and their images captured in a HCS system coupled with a 10x,^75^ ^,^ ^76^ 20x (most cases^60^ ^,^ ^73^ ^,^ ^77^ ^,^ ^78^) or 40x^67^ ^,^ ^75^ objective lens (Fig. 3A). Image analysis is performed using a custom pipeline in a proprietary e.g., Operetta Imaging System Harmony Software, PerkinElmer^77^ ^,^ ^79^ or free software e.g., Cellprofiler^58^ ^,^ ^80^. From this analysis, it is possible to obtain a few metrics used to measure compound antiparasitic activity, such as the number of amastigotes per cell and the percentage of infected cells.^69^ ^,^ ^73^ Additionally, an estimation of compounds’ cytotoxicity can be obtained in the same assay by counting the number of host cells i.e., their nuclei^69^ ^,^ ^77^ ^,^ ^81^ ^,^ ^82^ (Fig. 3B). In contrast to visual scoring, HCS-based assays are highly objective, accurate and faster, which make them an ideal tool for screening campaigns.^25^ Therefore, intracellular amastigote-based assays performed in HCS systems can become the new gold standard in trypanosomatid phenotypic screening by combining the necessary biological complexity of microscope-based assays with the high-throughput of microplate readers.

**Fig. 3:**
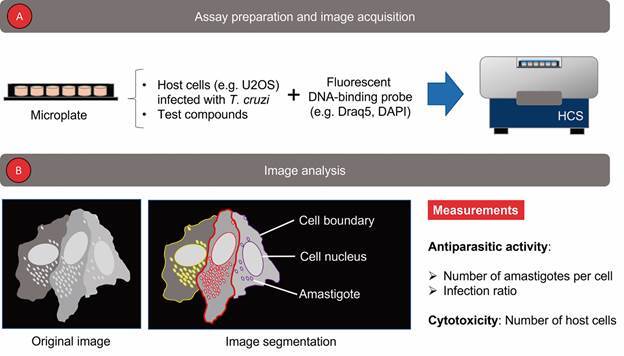
a single-probe HCS-based intracellular amastigote assay typically used in the search for new anti-*Trypanosoma cruzi* drugs. (A) Initially, the host infected cells treated with test compounds are stained with a fluorescente DNA-binding probe and their images captured by a HCS system. (B) During image analysis, cells and parasites are segmented individually and counted. From this it is possible to calculate at least two metrics related to compounds anti-trypanosomatid activity: number of amastigotes per cell and the percentage of infected cells (infection ratio). In addition, compounds cytotoxicity can be estimated from cells nuclei count.

**TABLE:** HCS-based assays employed in trypanosomatid drug discovery

Genus	Species	Strains	Evolutionary forms	Reporter	Host cell	Main measurements	Assay principle	Screened compounds	Pipeline step	References
*Trypanosoma*	*T. brucei brucei* (wild type and genetically engineered kDNA-independent cell line)^101^	Laboratory strain: Lister 427	Bloodstream	None	-	kDNA/nucleus ratio	Parasite nuclear DNA and kDNA detected by Hoescht 33342 while viable parasites by CFDA-SE	13,486 compounds from three commercial libraries (Prestwick Chemical Library, Screen-Well PKE library and BioAscent 12,000 diverse chemical libraries)	Primary screening	^(80)^
	*T. cruzi*	Laboratory strain: Y	Intracellular amastigotes	None	Human induced pluripotent stem cell-derived cardiomyocytes (hiPSC-CM)	Number of host cells (lethal cytotoxicity evaluation), number of amastigotes per cell, infection ratio and sublethal cytotoxicity-related parameters	Screening: Parasites DNA spots and cell nucleus detected by Draq5 while cell cytoplasm by cTNT immunostaining; Cytotoxicity assay: Cell nucleus, cytoskeleton and mitochondria detected by DAPI, Phalloidin-488 and MitoTracker^TM^ Red, respectively	4 compounds with known anti-trypanosomatid activity	Primary screening (dose-response) and cytotoxicity assay	^(71)^
		Laboratory strain: Tulahuen	Intracellular amastigotes	*Escherichia coli* beta-galactosidase gene^(44)^	Rat cardiomyocytes (H9c2)	Number of host cells (cytotoxicity evaluation), number of amastigotes per cell and percentage of infected cells per well	Parasites DNA spots as well as cell nucleus and cytoplasm detected by Draq5	20 compounds with known anti-trypanosomatid activity	Primary screening (dose-response) and cytotoxicity evaluation performed in the same assay	^(69)^
		Laboratory strain: Tulahuen	Intracellular amastigotes	None	Mouse embryo fibroblast (3T3)	Number of amastigotes per cell and number of infected cells per well	Parasites DNA spots and cell nucleus detected with Hoescht 33342 while cell cytoplasm with HCS CellMask Green^TM^	741 compounds (FDA-approved and with biological activity) from a in-house library and 685 compounds from a pilot collection of Medicines for Malaria Venture Malaria Box	Primary screening and dose-response assay	^(79)^
		Laboratory strain: Tulahuen	Intracellular amastigotes	None	Mouse embryo fibroblast (3T3)	Number of host cells (cytotoxicity evaluation), number of amastigotes per cell and number of infected cells per well	Parasites DNA spots and cell nucleus detected with Hoescht 33342 while cell cytoplasm with HCS CellMask Green^TM^	24,993 compounds optimised for lead-like properties	Primary screening and cytotoxicity evaluation performed in the same assay. A similar method was used to generate the dose-response curves and for further profiling of selectively active compounds. Protocol based on a previous report^(79)^. A wash-off assay was also carried out by HCS.	^(72)^
*Trypanosoma*		Laboratory strains: Silvio X10/7 subclone A1, Y, M6241 Clone 6, ERA Clone 2, PAH179 Clone 5, Tula Clone 2 and CL Brener.	Trypomastigotes and intracellular amastigotes	CL Brener strain carrying a gene reporter (red-shifted luciferase^102^)	*Cercopithecus aethiops* kidney cell (Vero)	Number of host cells (cytotoxicity evaluation), number of amastigotes per cell, number of amastigotes per well, percentage of infected cells and percentage of EdU (nucleoside analog) positive cells and parasites	Primary screeninging and replication assay (amastigotes): parasites DNA spots as well as cell nucleus and cytoplasm detected by Hoechst 33342; Proliferation assay : EdU incorporation by parasites (trypomastigotes and amastigotes) and cell nucleus detected by Click-iT Plus EdU Alexa- Fluor 488 Imaging Kit. Trypomastigote flagellum was also detected by immunostaining (anti-PFR1 antibody)	3 trypanocidal drugs	Primary screening (dose-response) and cytotoxic evaluation performed in the same assay. Replication and proliferation assays were also carried out using a HCS system. Protocol based on a previous report^(94)^.	^(73)^
		Not declared	Intracellular amastigotes	None	Human osteosarcoma cells (U2OS)	Number of host cells (cytotoxicity evaluation), number of amastigotes per cell and infection ratio	Parasites DNA spots as well as cell nucleus and cytoplasm detected by Draq5	2 trypanocidal drugs	Primary screening (dose-response) and cytotoxicity evaluation in the same assay	^(100)^
		Laboratory strains: Dm28c, Y, ARMA13 cl1, ERA cl2, 92-80 cl2, CL Brener and Tulahuen	Intracellular amastigotes	None	Human osteosarcoma cells (U2OS)	Number of amastigotes per cell and infection ratio	Parasites DNA spots as well as cell nucleus and cytoplasm detected by Draq5	8 lead/clinical compounds with anti-trypanosomatid activity	Primary screening (dose-response) and time-kill assay. Protocols based on a previous report^(100)^.	^(70)^
		Laboratory strain: Y	Intracellular amastigotes	None	Human osteosarcoma cells (U2OS)	Number of host cells (cytotoxicity evaluation), number of amastigotes per cell and infection ratio	Parasites DNA spots as well as cell nucleus and cytoplasm detected by Draq5	39 commercial compounds selected from a virtual screening of the ChemBridge chemical database (1 M compounds)	Primary screening (dose-response) and cytotoxicity evaluation performed in the same assay. Protocol based on a previous report^(70)^.	^(81)^
		Laboratory strains: Y-H10, Sylvio X10/1 and CL Brener	Intracellular amastigotes	None	Human osteosarcoma cells (U2OS), human acute monocytic leukemia (THP-1) cells, *Cercopithecus aethiops* kidney cell (Vero) and rat skeletal myoblast (L6) cells	Number of host cells (cytotoxicity evaluation), number of amastigotes per cell and infection ratio	Parasites DNA spots as well as cells nucleus and cytoplasm detected by Draq5	1,280 compounds from LOPAC library (Sigma-Aldrich)	Primary screening and cytotoxicity evaluation performed in the same assay using Y-H10 strain. Similar method was employed to dose-response curves. Protocol based on a previous report ^(93)^	^(27)^
		Laboratory strains: Y	Intracellular amastigotes	None	Human osteosarcoma cells (U2OS)	Number of host cells (cytotoxicity evaluation) and infection ratio	Parasites DNA spots as well as cells nucleus and cytoplasm detected by Draq5	24 novel compounds derived from farnesyltransferase inhibitors	Primary screening (dose-response) and cytotoxicity evaluation performed in the same assay . Protocol based on a previous report^(70)^),	^(93)^
		Laboratory strains: Y	Intracellular amastigotes	None	Murine fibroblasts (NIH 3T3) expressing GFP	Number of amastigotes per cell and percentage of infected cells	Parasites DNA spots and cells nucleus detected by DAPI while cell body detected by GFP	21 treatment groups from an on-going study	Primary screening	^(25)^
*Trypanosoma*		Laboratory strain: CA-I/72	Intracellular amastigotes	None	Mouse myoblasts (C2C12)	Number of host cells (cytotoxicity evaluation) and infection level (number of amastigotes per cell as determined by nuclei counting)	Parasites DNA spots and cells nucleus detected by DAPI	7,680 compounds from ReFRAME library	Primary screening and cytotoxicity evaluation performed in the same assay. A similar method was used to generate the dose-response curves. Protocol based on previous reports^(86,87,100)^	^(85)^
		Laboratory strains: CA-I/72	Intracellular amastigotes	None	Mouse myoblasts (C2C12)	Number of host cells (cytotoxicity evaluation) and infection level (number of amastigotes per cell)	Parasites DNA spots and cells nucleus detected by DAPI	Gallinamide A and 15 analogs	Primary screening (dose-response) and cytotoxic evaluation performed in the same assay. Protocol based on a previous report^(87)^	^(86)^
		Laboratory strain: CA-I/72	Intracellular amastigotes	None	Mouse myoblasts (C2C12)	Number of host cells (cytotoxicity evaluation) and infection ratio	Parasites DNA spots and cell nucleus detected by DAPI	97 commercial compounds selected by virtual screening	Primary screening and cytotoxicity evaluation performed in the same assay. A similar method was used to generate the dose-response curves	^(87)^
		Laboratory strain: Silvio X10/7 A1	Intracellular amastigotes	None	*Cercopithecus aethiops* kidney cells (Vero)	Number of host cells (cytotoxicity evaluation), number of amastigotes per cell and percentage of infected cells	Parasites DNA spots as well as cells nucleus and cytoplasm detected by Hoechst 33342	14,080 commercial compounds from NIH (clinical collection) and Selleck-Chem (FDA-approved drug library) libraries	Primary screening and cytotoxicity evaluation performed in the same assay. Similar methods were used to dose-response, cell replication, static-cidal and rate-of-kill assays. Protocols based on previous reports^(25,53,69,89)^.	^(94)^
		Laboratory strains: CA-I/72, PSD-1 and Sylvio X10/7	Intracellular amastigotes	None	Bovine embryo skeletal muscle (BESM) and human hepatoma (Huh-7) cells	Number of host cells (cytotoxicity evaluation) and kDNA/host nuclei ratio	Parasites kDNA and cell nucleus detected by DAPI	909 clinical compounds library from Iconix Biosciences	Primary screening and cytotoxicity evaluation performed in the same assay. A similar method was used to generate the dose-response curves	^(89)^
		Laboratory strain: CA-I/72	Intracellular amastigotes	None	Mouse myoblasts (C2C12)	Number of host cells (cytotoxicity evaluation), infection ratio and area of infection (area of kinetoplastids/total nuclei)	Parasites DNA spots and cell nucleus detected by DAPI, cell body inferred by aggregating parasites DNA spots and cell nucleus image objects	180,329 compounds from GNF Academic Collaboration Library	Primary screening and cytotoxicity evaluation performed in the same assay. A similar method was used to generate the dose-response curves. Protocols based on a previous report^(89)^	^(88)^
		Laboratory strain: Tulahuen	Intracellular amastigotes	*Escherichia coli* β-galactosidase gene^(44)^	Rat cardiomyocytes (H9c2)	Number of host cells (cytotoxicity evaluation), number of amastigotes per cell and infection ratio	Parasites DNA spots as well as cell nucleus and cytoplasm detected by Draq5	2,310 compounds from GlaxoSmithKline HTS screening collection	Orthogonal assay (screening and cytotoxicity evaluation). Protocol based on a previous report^(69)^	^(60)^
		Laboratory strain: STIB980 clone 1	Intracellular amastigotes	eGFP	Peritoneal mouse macrophages	Number of host cells (cytotoxic evaluation), number of amastigotes per image and fold change in parasite numbers per hour	Parasite kDNA and cell nucleus detected by Hoechst 33342. Parasites also identified by GFP staining in live imaging assay	2 anti-trypanosomatid drugs	Secondary assay (dose-response and cytotoxicity evaluation)	^(97)^
*Trypanosoma*		Laboratory strain: Tulahuen	Intracellular amastigotes	*Escherichia coli* β-galactosidase gene^(44)^	Rat cardiomyocytes (H9c2)	Number of host cells (cytotoxicity evaluation), number of amastigotes per cell and infection ratio	Parasites DNA spots as well as cell nucleus and cytoplasm detected by Draq5	3,598 compounds from Calibr Diversity Library	Secondary assay (screening and cytotoxicity evaluation) and dose-response assay. Protocol based on previous reports^(60,69)^	^(84)^
		Laboratory strain: Talahuen	Intracellular amastigotes	GFP^103^	Monkey kidney epithelial (LLCMK2) cells	Number of intracellular amastigotes per well	Cells nucleus detected by DAPI and amastigotes by GFP staining	4 phenothiazinium dyes	Primary screening (dose-response)	^104^
		Laboratory strain Tulahuen	Intracellular amastigotes	None	Mouse embryo fibroblasts (3T3)	Number of amastigotes per cell and number of infected host cells per well	Parasites DNA spots and cell nucleus detected by Hoescht 33342 while cell cytoplasm by HCS CellMask Green™	472 compounds from Davis open access natural product-based library	Primary screening and dose-response assay. Protocol based on a previous report^(79)^)	^(98)^
*Leishmania*	*L. donovani, L. amazonensis and L. braziliensis*	Laboratory strains: *L. donovan*i (MHOM/IN/1980/DD8), *L. amazonensis* (MHOM/BR/1977/LTB0016) and *L. braziliensis (MHOM/BR/1975/M2903)*	Intracellular amastigotes	None	Human acute monocytic leukemia (THP-1) cells	Number of host cells (cytotoxicity evaluation), number of amastigotes per cell and infection ratio	Parasites DNA spots as well as cell nucleus and cytoplasm detected by Draq5	1,280 compounds from LOPAC library	Primary screening and cytotoxicity evaluation performed in the same assay. A similar method was used to generate the dose-response curves. Protocol based on a previous report^(82)^	^(83)^
	*L. donovani*	Laboratory strain: MHOM/SD/62/1SCL2D, LdBOB	Intracellular amastigotes	GFP^(53)^	Human acute monocytic leukemia cells (THP-1)	Number of amastigotes per cell, infection ratio and number of parasites labeled with EdU	Cell nucleus and cytoplasm detected by DAPI while amastigotes by GFP staining. In the proliferation assay the incorporation of EdU in newly synthetized DNA was measured using Click-iT^TM^ Plus Alexa Fluor^TM^ 647 Picolyl Azide Toolkit	2 antileishmanial drugs	Primary screening (dose-response) and proliferation assay. Protocol based on previous reports^(53,60)^)	^(74)^
	*L. donovani*	Laboratory strain: MHOM/SD/62/1S-cl2D	Intracellular amastigotes	None	Human acute monocytic leukemia (THP-1) cells	Number of host cells (cytotoxicity evaluation) and number of amastigotes per cell	Parasites kDNA and cell nucleus detected by DAPI. Cell boundary was delineated around the nucleus object.	909 bioactive compounds library from Iconix Biosciences	Primary screening and cytotoxicity evaluation performed in the same assay. A similar method was used to generate the dose-response curves	^(96)^
	*L. donovani, L. amazonensis, L. braziliensis,* *L. major*	Laboratory strains: *L. donovani* (MHOM/ET/67/HU3), *L. amazonensis* (MHOM/BR/73/M2269), *L. braziliensis* (MHOM/BR/ 2903) and *L. major* (MHOM/IL/81/FRIEDLIN)	Intracellular amastigotes	None	Human acute monocytic leukemia cells (THP-1)	Number of host cells (cytotoxicity evaluation), number of amastigotes per cell and infection ratio	Parasites DNA spots and cell nucleus detected by Draq5. Individual cells were segmented after a series of computational tasks that use the nucleus as a seed point	4 antileishmanial drugs	Primary screening (dose-response) and cytotoxicity evaluation performed in the same assay	^(82)^
*Leishmania*	*L. donovani and L. major*	Laboratory strains: *L. donovani* (MHOM/ ET/67/HU3 ) and *L. major* (MHOM/IL/81/FRIEDLIN)	Intracellular amastigotes	None	Human acute monocytic leukemia cells (THP-1)	Infection ratio	Parasites DNA spots as well as cell nucleus and cytoplasm detected by Draq5	124 compounds from TimTec library	Hit confirmation assay (dose-response)	^(68)^
	*L. donovani*	Laboratory strain: MHOM/SD/62/1S-CL2D (LdBOB)	Intracellular amastigotes	*Aequorea victoria* eGFP	Human acute monocytic leukemia cells (THP-1)	Number of host cells (cytotoxicity evaluation), number of amastigotes per cell and number of infected cells	Cell nucleus and body detected by DAPI and HCS Cellmask^TM^ Deep Red, respectively. Parasites detected by GFP staining	15,659 diverse compounds library	Primary screening and cytotoxicity evaluation performed in the same assay. A similar method was used to generate the dose-response curves	^(53)^
	LRV1-containing *L. guyanensis*	Laboratory strain: MHOM/ BR/75/M4147	Intracellular amastigotes	None	Primary murine bone-marrow derived macrophages	Number of host cells (cytotoxicity evaluation) and number of amastigotes per cell	Parasites DNA spots and cells nucleus detected by DAPI while cell cytoskeleton (cytosol) by phalloidin-Alexa488	1,520 compounds from Prestwick Chemical Library	Primary screening and cytotoxicity evaluation performed in the same assay. A similar method was used to generate the dose-response curves. Protocol based on a previous report^(76)^	^(57)^
	*L. infantum* and *L. amazonensis*	Laboratory strains: *L. infantum* (MHOM/MA/67/ITMAP-263) and *L. amazonensis* (MHOM/ BR/LTB0016)	Intracellular amastigotes	None	Primary murine bone-marrow derived macrophages	Number of amastigotes per cell and percentage of infected cells	Parasites DNA spots and cells nucleus detected by DAPI while cell body by CellMask^TM^ Deep Red	1 antileishmanial drug	Primary screening (dose-response). Protocol based on previous reports^(53,57,82,96)^	^(58)^
	*L. donovani and L. amazonensis*	Laboratory strains: *L. donovani* (MHOM/SD/62/1S-CL2D) and *L. amazonensis* (MPRO/BR/1972/M1841)	Intracellular amastigotes	mCherry (*L. amazonensis*)	Primary murine bone-marrow derived macrophages	*L. donovani*: Number of host cells (cytotoxicity evaluation), number of amastigotes per cell and percentage of infected cells; *L. amazonensis*: Number of amastigotes, number of total host cells (TM, nucleus counting), number of healthy host cells (HM, based on nucleus size and intensity features), number of parasitophorous vacuoles (PV), viability index (HM/TM) and PV/HM ratio	*L. donovani* assay: parasites DNA spots and cell nucleus detected by Hoescht 33342 while cell body by HCS CellMask^TM^ Blue. Parasites detected by immunostaining using hamster (infected with *L. donovani)* immune serum and secondary antibody anti-hamster conjugated with Alexa Fluor 488; *L. amazonensis* assay: cell nucleus, parasitophorous vacuoles and parasites detected by Hoescht 33342, LysoTracker Green DND-26 and mCherry staining, respectively	188 compounds from Leish-Box	Primary screening and cytotoxicity evaluation performed in the same assay (*L.donovani* and *L. amazonensis*). A similar method was used to generate the dose-response curves (*L. donovani*). Protocol based on a previous report^(76)^	^(75)^
*Leishmania*	*L. major, L. donovani* and *L. infantum*	Laboratory strains: *L. major* (MHOM/SU/73/5ASKH), *L. donovani* (BPK190), *L. infantum* (MHOM /FR/91/LEM 2259, belonging to zymodeme MON-1 clone 3511) and *L. braziliensis* (MHOM/PE/01/PER005 cl.2)	Intracellular amastigotes	None	Primary murine bone-marrow derived macrophages	Number of host cells (cytotoxicity evaluation), number of amastigotes per well, number of amastigotes per cell and infection ratio	Cell nucleus detected by DAPI while cell cytoplasm and parasites detected by immunostaining using mouse anti-Hsp9 primary antibody and anti-mouse secondary antibody conjugated with Alexa Fluor 647	6 immunostimulatory *Eh*PIb-compounds	Primary screening (dose-response) and cytotoxicity evaluation performed in the same assay	^(77)^
	*L. amazonensis*	LV79 (MPRO/BR/1972/M1841)	Intracellular amastigotes	*Ds*Red2^105^	Primary murine bone-marrow derived macrophages	Number of amastigotes, number of total host cells (TM, nucleus counting), number of healthy host cells (HM, based on nucleus size and intensity features), number of parasitophorous vacuoles (PV), viability index (HM/TM) and PV/HM ratio	Cell nucleus, parasitophorous vacuoles and parasites detected by Hoescht 33342, LysoTracker Green DND-26 and *Ds*Red2 staining, respectively	60 compounds with established or potential leishmanicidal, antifungal or antimicrobial and cytotoxic activities	Primary screening and cytotoxicity evaluation performed in the same assay. A similar method was used to generate the dose-response curves	^(76)^
	*L. mexicana*	MNYC/BZ/62/ M379	Intracellular amastigotes	None	Human acute monocytic leukemia cells (THP-1)	Number of host cells (cytotoxicity evaluation), average number of amastigotes per cell and frequency distribution of intracellular amastigotes	Parasites detected by CellTracker^TM^ Orange CMRA while cell body and nucleus detected by CellTracker^TM^ Green CMFDA and DAPI, respectively	3 antileishmanial drugs	Primary screening (dose-response) and cytotoxicity evaluation performed in the same assay	^(78)^
	*L. donovani*	Recent clinical isolates sensitive and resistant to pentavalent antimonials (SSG):SSG-sensitive (MHOM/NP/03/BPK282/0 clone 4) and SSG-resistant (MHOM/NP/03/BPK275/0 clone 18)	Intracellular amastigotes	None	Human acute monocytic leukemia cells (THP-1)	Number of amastigotes per cell and infection ratio	Parasites DNA spots as well as cells nucleus and cytoplasm detected by Draq5	130 compounds from Leish-box (GSK)	Primary screening (dose-response). Protocol based on previous reports^(53,60)^	^(67)^
	*L. donovani*	Laboratory strain: MHOM/SD/62/1S-CL2D	Intracellular amastigotes	None	Human acute monocytic leukemia cells (THP-1)	Number of host cells (cytotoxicity evaluation), number of amastigotes per cell and infection ratio	Parasites DNA spots as well as cell nucleus and cytoplasm detected by Draq5	1,742 bioactive compounds from MedChem Express	Primary screening and cytotoxicity evaluation performed in the same assay. A similar method was used to generate the dose-response curves. Protocol based on a previous report^(82)^	^(95)^
	*L. donovani*	Laboratory strain: MHOM/SD/62/1S-CL2D, LdBOB	Intracellular amastigotes	*Aequorea victoria* eGFP	Human acute monocytic leukemia cells (THP-1)	Number of host cells (cytotoxicity evaluation), number of amastigotes per cell and infection ratio	Cell nucleus and cytoplasm detected by DAPI while parasites by GFP staining	32,200 compounds from GlaxoSmithKline HTS screening collection	Secondary screening and dose-response assay (also cytotoxicity evaluation). Protocol based on previous reports^(53,76)^	^(60)^
*Leishmania*	*L. donovani*	Laboratory strain: MHOM/SD/62/1S-CL2D, LdBOB	Intracellular amastigotes	*Aequorea victoria* eGFP	Human acute monocytic leukemia cells (THP-1)	Number of host cells (cytotoxicity evaluation), number of amastigotes per cell and infection ratio	Cell nucleus and cytoplasm detected by DAPI while parasites by GFP staining	1,392 compounds from Diversity Library from Calibr	Secondary screening and cytotoxicity evaluation performed in the same assay. A similar method was used to generate the dose-response curves. Protocol based on previous reports^(60,74)^)	^(84)^
	L. donovani	Laboratory strain: MHOM/IN/80 DD8	Intracellular amastigotes	None	Human acute monocytic leukemia cells (THP-1)	Number of amastigotes per cell and number of infected cells normalized to the positive and negative controls	Parasites DNA spots detected by SYBR green while cell body and nucleus by CellMask^TM^ Deep Red and SYBR green, respectively	472 natural product-derived library from Davis open access	Primary screening and dose-response assay. Protocol based on a previous report^(99)^	^(98)^
	*L. donovani*	Laboratory strain: MHOM/IN/80 DD8	Intracellular amastigotes	None	Human acute monocytic leukemia cells (THP-1)	Number of amastigotes per cell and number of infected cells normalized to the positive and negative controls	Parasites DNA spots detected by SYBR green while cell body and nucleus by CellMask^TM^ Deep Red and SYBR green, respectively	400 compounds from Medicines for Malaria Venture Pathogen Box	Primary screening and dose-response assay	^(99)^

CFDA-SE - 5(6)-carboxyfluorescein diacetate succinimidyl ester, CMFDA - 5-chloromethylfluorescein diacetate, cTNT - cardiac troponin-T, DAPI - 4′,6-diamidino-2-phenylindole, EdU - 5-ethynyl-2-deoxyuridine, GFP - Green fluorescent protein, GNF - Institute of the Novartis Research Foundation, Hsp90 - 90 kDa heat shock protein, HTS - High throughput screening, kDNA - Kinetoplast DNA, LOPAC - Library of Pharmacologically Active Compounds, LRV1 - Leishmania RNA virus 1, PFR1 - paraflagellar rod protein 1, ReFRAME - Repurposing, Focused Rescue, and Accelerated Medchem.

A variety of trypanosomatid species and/or strains have been interrogated in HCS-based assays (Table). Most of the studies with *Trypanosoma* spp. were carried out with strains of *T. cruzi* from different DTUs. Likewise, many strains of both dermatropic and viscerotropic *Leishmania* species have been investigated. In most cases, they consist of laboratory strains which are easily cultivated *in vitro*. Nonetheless, the solely use of these strains demand caution since they may differ from clinical isolates in terms of genotype and phenotype (e.g., drug resistance).^17^ ^,^ ^67^ Therefore, it is advisable to confirm hit compounds on a panel of clinical isolates and groups of representative strains (e.g., for each DTU).^17^ ^,^ ^18^ ^,^ ^67^ ^,^ ^70^ ^,^ ^83^

The selection of a disease-relevant cell host model at the initial stages of the drug discovery pipeline is crucial to reduce the attrition rates with later steps. The HCS-based methods proposed so far for trypanosomatids were developed using primary cells or commercial cell lines from human or other organisms (murine and primate) sources (Table). Most *T. cruzi* screenings measured the antiparasitic activity of compounds in muscle cells infected with amastigote forms.^27^ ^,^ ^60^ ^,^ ^69^ ^,^ ^71^ ^,^ ^84^ ^-^ ^89^ They represent a particularly good model for compound screening since the pathology described in the chronic phase of Chagas disease is mainly related to the presence of *T. cruzi* in these cells and they have a high susceptibility to infection *in vitro*.^69^ Some reports show that compound activity may vary between different cell types suggesting the presence of specific host-parasite interactions.^27^ ^,^ ^90^ Therefore, it is suggested that a given hit should be tested against multiple cell models to confirm its potential anti-*T. cruzi* activity.^27^

For *Leishmania* spp., HCS-based drug screening has been carried out with macrophages or macrophage-like cells: primary murine bone-marrow derived macrophages (BMDM) and human acute monocytic leukemia cells (THP-1), a commercial cell line. Macrophages are disease-relevant models since they exert a dual role in leishmaniasis being at the same time the final host cell for parasite proliferation and the effector cell that contributes to clean the infection.^91^ Most assays use THP-1 cells instead of primary cells due to several technical and logistic advantages of the former, such as lower cost, ease of cultivation, applicability to large screening campaigns and less ethical restriction.^77^ ^,^ ^92^ However, they require external chemical stimuli for monocyte to macrophage transformation and show much less biological relevance.^76^ ^,^ ^92^ In this context, it is advisable to test the compounds in primary cells whenever possible.^53^ ^,^ ^92^

Different methods have been proposed to quantify the number of trypanosomatid amastigotes inside the host cell in HCS-based assays (Table). A common approach consists of using a single DNA-binding fluorescent probe (e.g., Draq5) to stain both cells (nuclei and/or cytoplasm) and parasites (DNA spots composed of kDNA and/or nuclear DNA)^67^ ^,^ ^68^ ^,^ ^69^ ^,^ ^73^ ^,^ ^83^ ^,^ ^87^ ^,^ ^93^ ^-^ ^95^ (Fig. 3). In this strategy, all objects necessary for calculating the antiparasitic activity of a test compound are contained in the same image. During analysis, cell nuclei and parasite DNA spots are distinguished by size and counted. Cell boundaries can be revealed from probes “leakage” into the cytoplasm and/or inferred by computational tools, allowing the determination of the number of parasites per each cell.^67^ ^,^ ^82^ ^,^ ^95^ ^,^ ^96^ These methods are simple, but they may underestimate parasitaemia when parasites are located at the same place or near a cell’s nucleus, as well as detect non-specific stained spots due to the accumulation of host cell RNA in the cytosol.^25^ ^,^ ^69^ ^,^ ^77^ ^,^ ^78^ Moreover, they generally require cell fixation making it impossible to monitor live cells over time which could inform the time-course of drug action.^97^ The use of computer algorithms to delineate cell boundaries based on nucleus position may also be prone to errors since they often consider that the nucleus is located at the centre of each cell.^82^ ^,^ ^96^ This may not be true specially for primary cells once their morphology is not as homogeneous as that observed for cell lineages.^58^ ^,^ ^77^ Therefore, some authors have proposed more elaborated assays using multiple fluorescent probes, cells/parasites carrying reporter genes or immunostaining to better define each image object. The former strategy includes, per instance, the combination of a DNA binding probe (e.g., DAPI) to detect the parasite and another probe to stain the whole host cell or its cytoplasm (e.g., CellMask^TM^ dyes).^58^ ^,^ ^78^ ^,^ ^79^ ^,^ ^98^ ^,^ ^99^ Recently, transgenic parasites expressing reporter genes (e.g., GFP and mCherry) have also been employed in HCS-based assays and represent a useful way to facilitate their identification in the images and reduce assay cost.^12^ ^,^ ^60^ ^,^ ^74^ ^,^ ^75^ ^,^ ^84^ ^,^ ^97^ ^,^ ^100^ However, this approach has some important drawbacks. One of them is that the parasite carrying this gene is no longer wild-type, which may affect its drug response and its interaction with the host cell.^12^ ^,^ ^58^ ^,^ ^100^ Moreover, the technique used to incorporate it into the parasite must be performed for each new species or strain (e.g., a clinical isolate).^58^ Another approach consists in using immunostaining to detect the parasite,^75^ the host cell^71^ or both.^77^ In this technique, one or more e.g., immune serum^75^ antibodies directly bind to parasites/cell antigen(s), whereas a fluorescent-conjugated secondary antibody binds to the antigen-antibody complex allowing its detection. This strategy allows the use of the same image analysis protocol regardless of the parasite strain though it tends to be more time-consuming.^77^

Apart from infection-related metrics, HCS-based assays may also provide other valuable information about the effect of test compounds on the parasite-host interaction. Depending on the probe(s) used to stain the biological sample it is possible to extract and quantify a variety of phenotypic features from image objects, including whole organisms i.e., host cells^53^ and parasites^60^ and/or subcellular compartments e.g., cell nuclei,^89^ kDNA,^80^ pharmacophores vacuoles,^76^ cytoskeleton^71^ Certain features, such as those related to morphology (e.g., area, shape) may be altered in the presence of the compound, giving a more detailed description of its antiparasitic and/or cytotoxicity activities.^71^ ^,^ ^76^ ^,^ ^80^ Sometimes the alterations are not necessarily associated with morphological changes or be detectable by the probes available. In this case, the use of stains that are sensitive to parasite/cell metabolic activity may be useful as they are not constrained to a specific mechanism. As listed in Table, only two dyes have been successfully employed to detect viable trypanosomatids in HCS-based assays: CFDA-SE^80^ (bloodstream *T. brucei*) and CellTracker Orange CMRA^78^ (promastigote and amastigote of *L. mexicana*). In contrast, there are several viability assays, such as those designed for plate-readers (previously discussed in the text), that could be used as orthogonal methods for this kind of analysis.

*Successful examples of HCS-based assays in trypanosomatid screening campaigns* - Table shows several examples of HCS-based methods in trypanosomatid drug discovery. Some of them helped to reveal the antiparasitic activity of compounds in screening campaigns (Fig. 4). Bernatchez and colleagues,^85^ for instance, performed a primary screen of 7,680 compounds with confirmed clinical safety (ReFRAME library) on cells infected with *T. cruzi* amastigotes using a HCS-based assay. This technique allowed the identification of seven molecules with potent antiparasitic activity (EC_50_ values: 0.44 to 480 nM) and high selectivity index (≥ 10). One of the most promising compounds for drug development was 348U87 (**8**) (EC_50_: 0.63 nM and selectivity index: 1294), a small molecule with antiherpetic properties. Apart from primary screening, HCS technology has also been used in orthogonal/secondary assays.^60^ ^,^ ^68^ ^,^ ^84^ Peña and colleagues^60^ employed an interdisciplinary approach to detect potential anti-trypanosomatid agents in a 1.8 mi proprietary compounds (GlaxoSmithKline) library. The primary screening was carried out with microplate-based fluorometric assays using axenic (*L. donovani*) and intracellular (*T. cruzi*) amastigotes, as well as bloodstream (*T. brucei*) parasite forms. The resulting hits followed different paths in the drug discovery pipeline according to the species which included both experimental (e.g., cytotoxicity assay) and computational (e.g., physicochemical filters) steps. For *T. cruzi* and *L. donovani* HCS-based assays were also used to select active compounds against intracellular amastigotes. By the end of the pipeline, three sets (or “boxes’) of compounds with antiparasitic activity and low cytotoxicity were assembled: Leish-BOX (n = 192), Chagas-BOX (n = 222) and HAT-BOX (n = 192), which are provided as an open source for lead discovery programmes.

**Fig. 4:**
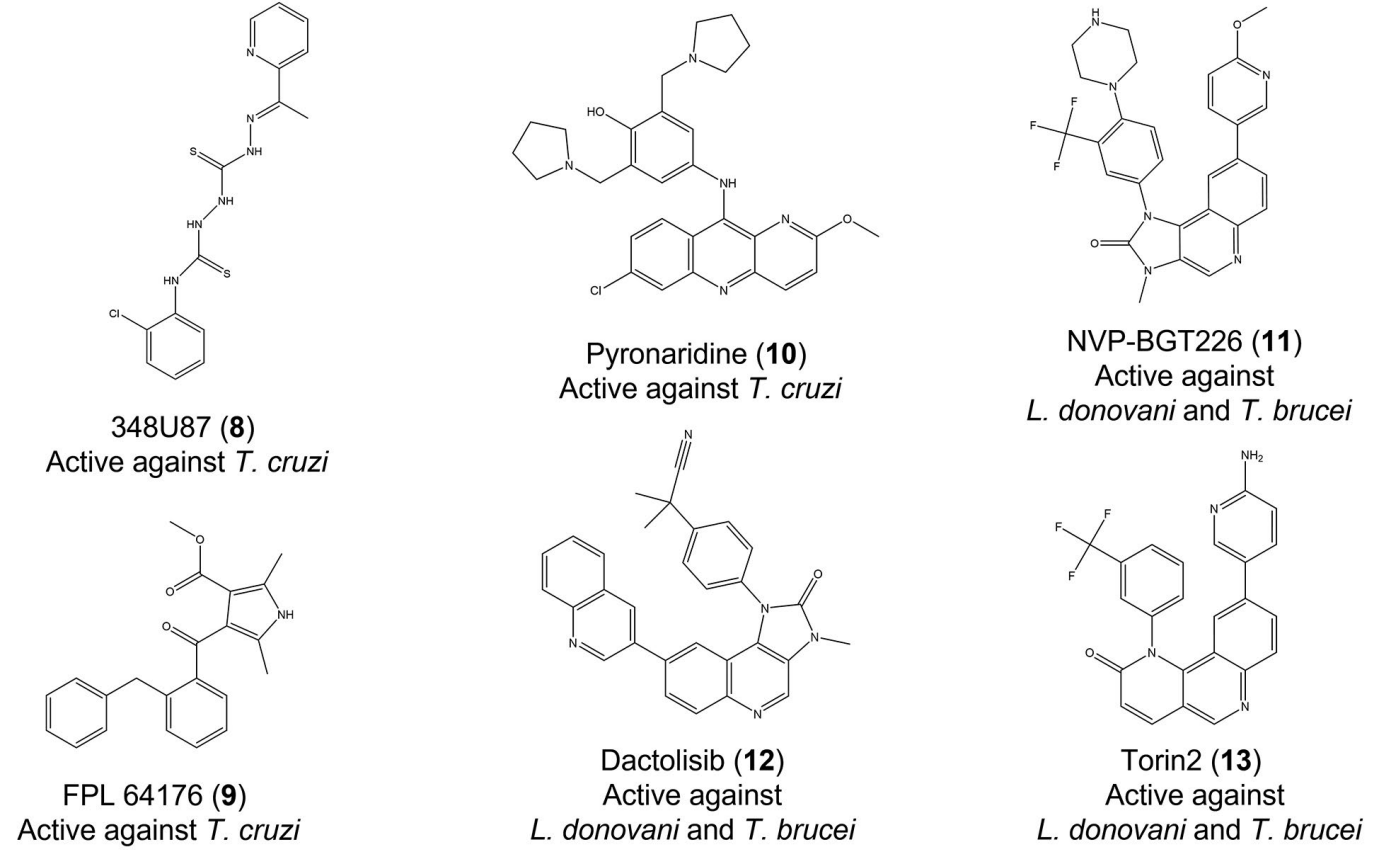
compounds with anti-trypanosomatid activity identified in screening campaigns that used HCS-based assays.

Though most assays use only one combination of parasite and host cell types, there are some exceptions.^27^ ^,^ ^77^ ^,^ ^83^ ^,^ ^89^ Franco and colleagues,^27^ for instance, conducted a primary screening consisting of four parallel HCS-based assays, one per host cell lineage. In each assay, 1,280 pharmacologically active compounds (LOPAC library, Sigma-Aldrich) were tested against cells infected with intracellular amastigotes of *T. cruzi* Y-H10 strain, yielding 82 unique hits. The compounds that were active in at least three cell lineages (n = 11) had their EC_50_ values calculated, which in most cases were at low micromolar range or lower. One of these compounds, FPL 64176 (**9**), a Ca^2+^ activator, was active on all four cell lines models and showed a similar potency (2.2 - 3.0 μM) across three parasite strains (Y-H10, Sylvio X10/1 and CL Brener), as well as good selectivity indexes (57.7, 35.4 and > 90, respectively) in U2OS cells.

A few reports have also validated the *in vitro* activity of test compounds on *in vivo* disease models.^87^ ^,^ ^88^ ^,^ ^95^ Ekins and colleagues^87^ measured the anti-trypanosomatid activity of commercial compounds combining computational and experimental techniques. Initially, they trained bayesian machine learning models to identify compounds with potential anti-*T. cruzi in vitro* activity. These models were then used to screen approximately 7,200 small molecules available in different chemical libraries (mostly from commercial sources). The 97 virtual hits with the highest scores were evaluated in a HCS-based assay that measured their antiparasitic activity and host cell toxicity. Dose-response curves revealed that five of them had an EC_50_ lower than 1 μM. Later, they had their *in vivo* efficacy determined using an acute Chagas mouse model. One of these compounds, pyronaridine (**10**) (antimalarial drug), had never been tested in a mouse model and showed a high efficacy (85.2 %) when compared to benznidazole (**1**) (100%). Some potential targets were predicted for this compound using different computational resources, including the *T. cruzi* pathway model developed in the same study. These results suggested that pyronaridine (**10**) may be a promising starting point for drug development. Phan and colleagues^95^ also obtained antiparasitic compounds with *in vitro* and *in vivo* activity against *L. donovani* and *T. brucei*. A primary screening of 1,742 commercial bioactive compounds (MedChem Express) was performed with a HCS-based assay using cells infected with intracellular amastigotes of *L. donovani*. This technique revealed 20 molecules with high antileishmanial activity and low cytotoxicity. Some of them were identified as inhibitors of the mammalian target of rapamycin (mTOR)/phosphoinositide 3-kinase (PI3K) (mTOR/PI3K) signaling pathway and had their EC_50_ values determined (0.14 - 13.44 μM). The three most potent molecules NVP-BGT226 (**11**), dactolisib (**12**) and Torin2 (**13**) were tested *in vivo* using a mouse model infected with *L. donovani*. They all inhibited parasitaemia in mice, especially NVP-BGT226 (**11**) (54 % inhibition). This compound also showed anti-*T. brucei in vitro* activity (resazurin-based assay) and reduced parasitaemia in a *T. brucei* infected mice model suggesting a broad anti-trypanosomatid effect. However, further studies may be necessary to increase their selectivity as mTOR and kinetoplastid TORs show high structural similarities and mTOR/PI3K inhibitors have already shown toxicity in clinical trials.

*An HCS-centered ideal assay cascade for antileishmanial phenotypic drug screening* - In screening campaigns, it is important to balance reliability and pragmatism (Fig. 5). Therefore, the use of *L. amazonensis* as a starting point is suggested, due to its rapid growth and high infectivity rate. Ideally, the reporter gene is integrated into the parasite’s DNA, to reduce some interference, such as variation in the number of plasmids per cell, or even the lack of it. In the hit discovery step, the use of cell lines such as J774 or THP-1 is acceptable to reduce the number of animals. However, at later stages, primary cells are recommended.^92^ Still considering pragmatism, the first round using only one concentration reduces resources and improves yield. Here, we adopted the limit of 10 µM, as recommended as a hit criterion by an expert panel.^106^ Another relevant point is to keep cells untreated for up to 24 hours after infection, to ensure complete transformation of promastigotes into amastigotes. Otherwise, the compounds can act on any remaining target of the promastigote. An advantage of the HCS is the possibility of obtaining cytotoxicity data together with the IC_50_ determination, allowing the disposal of toxic candidates early in the process. At later stages, it becomes necessary to use more clinically relevant *Leishmania* species, as well as to determine the CC_50_ in primary uninfected cells, before going to in vivo assays.

**Fig. 5:**
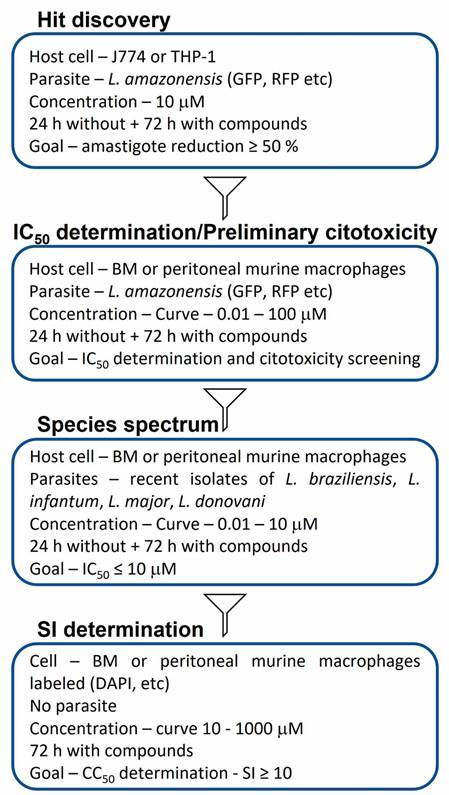
HCS-centered ideal assay cascade for antileishmanial phenotypic drug screening.

*Final remarks* - As discussed here, automated, image-based screening approaches known as HCS, have already made a major impact in trypanosomatid drug discovery and hold the promise to keep occupying an important space in the field for the years to come. This is so because HCS has several advantages over non image-based HTS including acquisition of multidimensional (2D and 3D) data, multiplexing capacity and multiparametric analysis for phenotypic scoring.

Even with the technological advantages of HCS, it´s key for the success of a trypanosomatid drug screening campaign to use carefully standardised reagents and optimised assays parameters. Compound-parasite incubation times and the sequential cell and compound seeding schemes have already been demonstrated as crucial factors that can be responsible for apparent lack of activity of compounds in a particular assay setup.^107^ Moreover, as a high-throughput experimental method, HCS presents a number of intrinsic challenges such as: experimental design errors, high cost and availability of materials (cells, proteins, compounds, etc.); compounds and reagents-related issues (incorrect structures, mixtures and salts, inconsistent batches, poor solubility); and other technical problems (pipetting errors and mechanical failures, temperature gradients, position effects, suboptimal readings). For instance, frequent hitter compounds (aggregators, interferers, etc.) may lead to false positives in screening campaigns and failure to validate initial hits on secondary assays.^108^ ^,^ ^109^

A better understanding of the host/parasite interaction and the disease itself is essential if we are to be able to design better and more predictive phenotypic assays. For instance, there are still unanswered questions regarding cell dormancy in *T. cruzi*: do we need assays targeting replicating and non-replicating forms of parasites?

A combination of phenotypic and target-based drug discovery approaches should lead to better chances of identifying compounds with the potential to satisfy the target product profile (TPP) of diseases caused by trypanosomatids. Additionally, the integration of machine learning and other AI or computational modeling techniques should help to make the most efficient use of resources.

Finally, incorporating newly developed assays into the phenotypic screening cascade is an exciting perspective to the field. Exploration of novel genetic editing methods, such as CRISPR/Cas9,^110^ allows creation of dual or even triple reporter systems for in *vitro* and *in vivo* multimodal imaging.^110^ ^,^ ^111^ These parasite cell lines enable efficient *in vivo* localisation and phenotyping, expanding the toolbox for trypanosomatid drug discovery.
